# Supplementary material for: Accurate predictive model of band gap with selected important features based on explainable machine learning
Source: Sci Rep. 2026 Apr 29;16:19850. doi: 10.1038/s41598-026-50566-3 (PMC13316055; doi:10.1038/s41598-026-50566-3)
Supplement: Supplementary file 1 — Supplementary Information. [file 41598_2026_50566_MOESM1_ESM.pdf]

Supplementary Information :

Accurate predictive model of band gap with selected important features based on explainable machine learning

Joohwi Lee[1*] and Kaito Miyamoto[1]

[1]Toyota Central R&D Labs., Inc., Yokomichi 41–1, Nagakute, Aichi, 480-1192, Japan.

*Corresponding author(s). E-mail(s): j-lee@mosk.tytlabs.co.jp;

Supplementary figures and tables

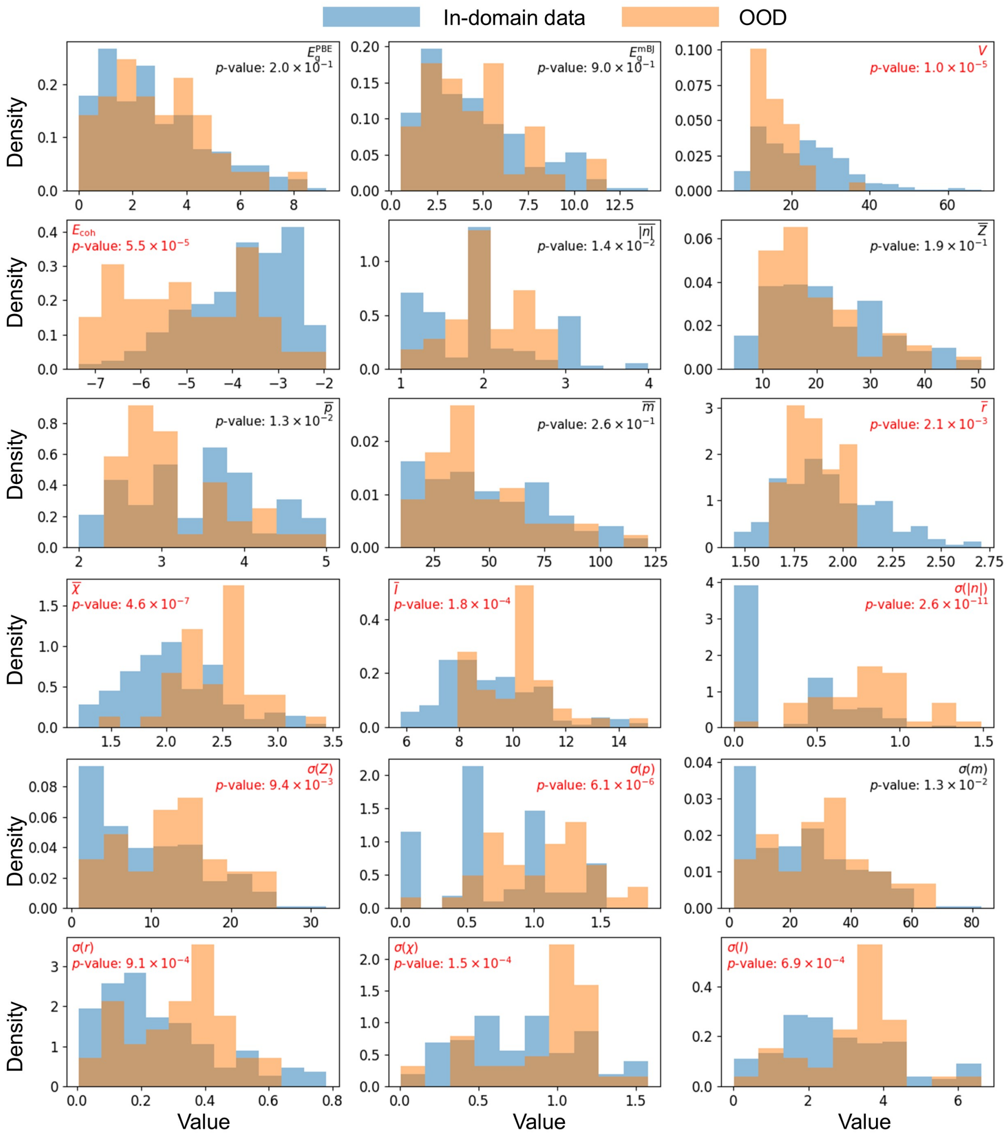

Fig. S1: Comparison of in-domain (270 compounds composed of binary and ternary systems) and out-of-domain (OOD) datasets (40 compounds containing transition metals and/or quaternary/pentanary systems) for 18 material features. Each subplot shows the probability density distributions of the corresponding feature for both datasets. The Kolmogorov–Smirnov test is used to determine whether the two distributions originate from the same population. The p-value is shown in each panel; values smaller than 0.01, shown in red, indicate that the in-domain and OOD distributions are significantly different at the 99% confidence level.

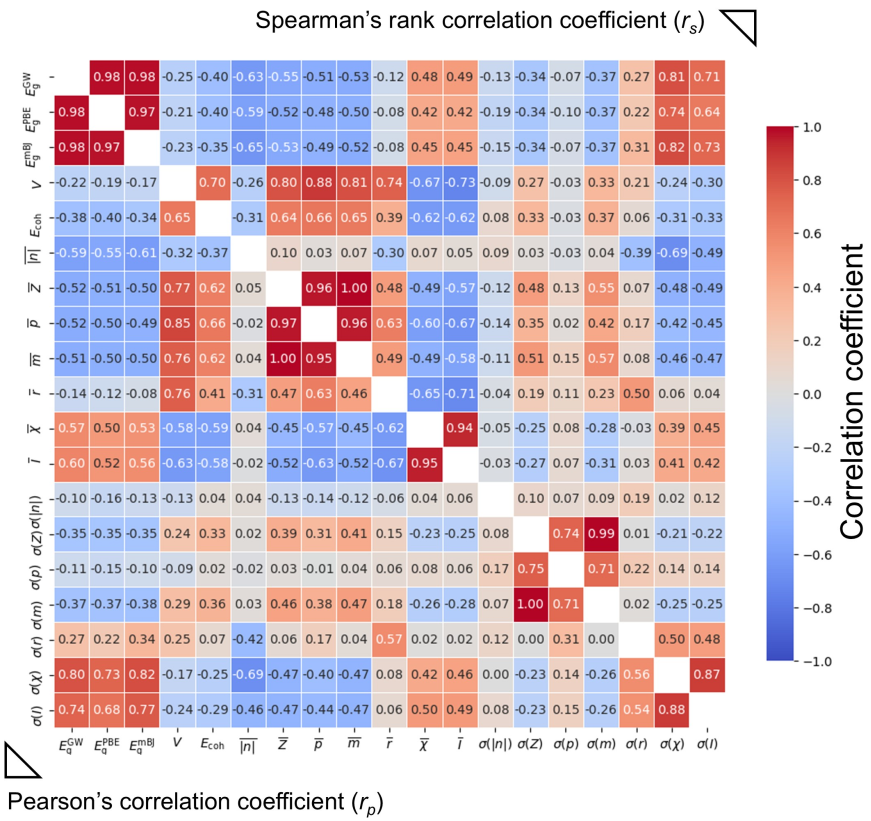

Fig. S2: Correlation coefficients for relationship between E_g^{GW} and 18 features for 270 binary and ternary inorganic compounds. The left-lower and right-upper triangles represent r_p and r_s, respectively.

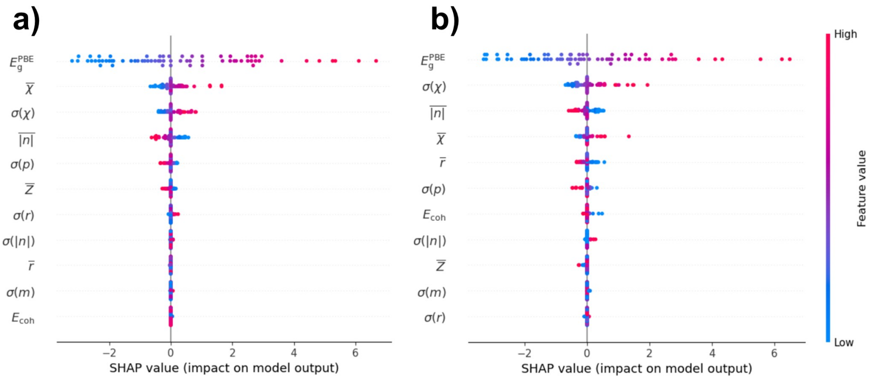

Fig. S3: Beeswarm plots from SHAP analysis using SVR model with *11-feature set* for predicting E_g^{GW}. The plots illustrate the relationships between the SHAP values and features in the test dataset, along with their distributions in panels (**a**) and (**b**), representing two different data samplings. Notably, for some features with low SHAP importance (indicated by small feature-distribution ranges), the impact of increases or decreases in the feature values on the SHAP values follows different trends for different samples.

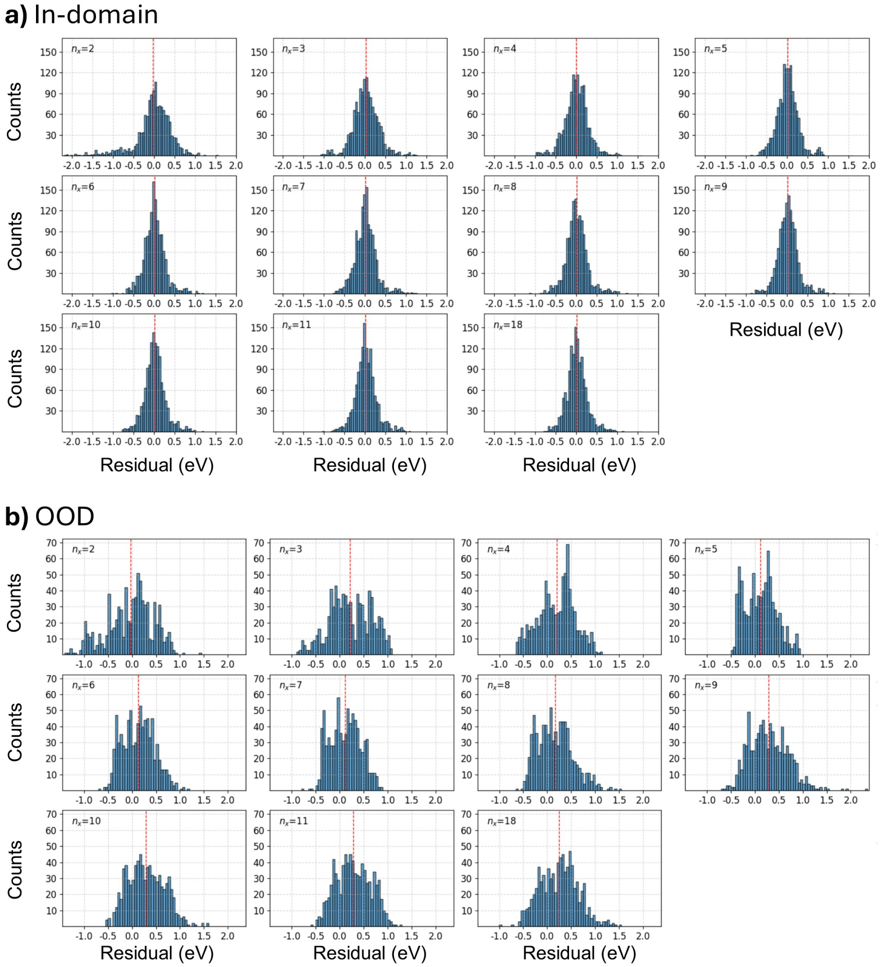

Fig. S4: Comparison of residual distributions of SVR models for (**a**) in-domain (20 data selections $\times$ 68 test data) and (**b**) OOD (20 data selections $\times$ 40 data) data across different number of features. The red, dashed, vertical line indicates the mean of the residual distribution for each model.

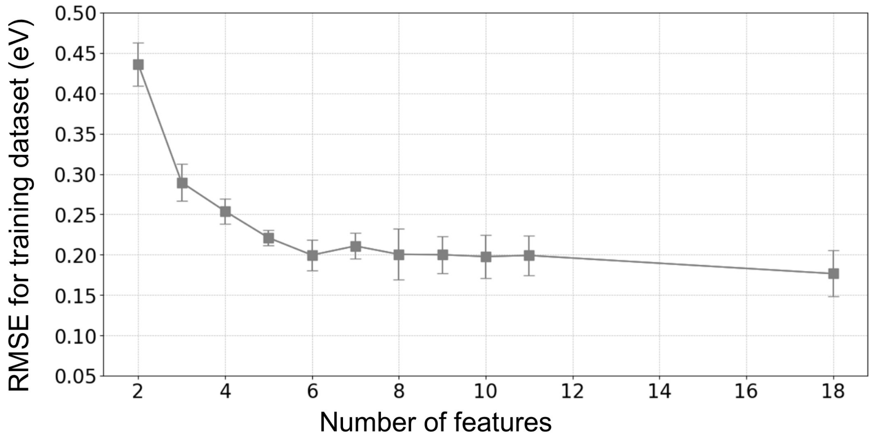

Fig. S5: Dependence of RMSE for the training dataset of SVR model on the number of features selected based on XML importance scores. The value at n_x = 18 corresponds to the pristine model. The error bars indicate one standard deviation across the predictive models with 20 different data selections.

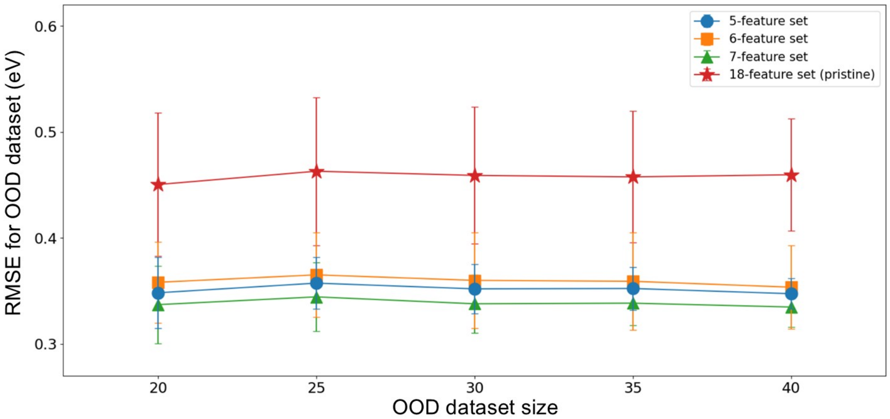

Fig. S6: Dependence of RMSE for the OOD dataset on size of OOD dataset. The value at $n_x = 18$ corresponds to the pristine model. The error bars indicate one standard deviation across the predictive models with 20 different data selections.

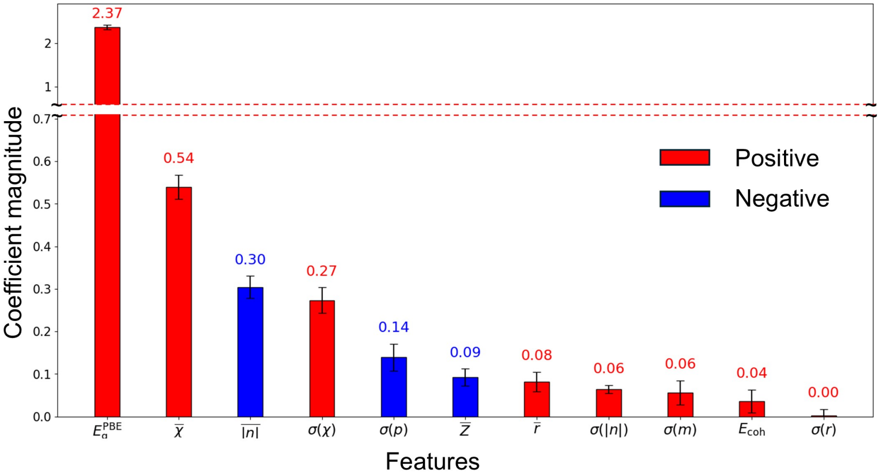

Fig. S7: Coefficient magnitudes of LASSO regression models for predicting $E_{\mathrm{g}}^{\mathrm{GW}}$ using the *11-feature* set. The absolute coefficient magnitudes serve as measures of feature importance in LASSO. The bars are colored red or blue when the coefficient sign remains consistently positive or negative, respectively, across the 20 predictive models. The error bars represent one standard deviation of the coefficient magnitudes across the predictive models constructed using 20 different data selections. The horizontal axis indicates the features ranked in descending order of their absolute coefficient magnitudes in the *11-feature* model, where the same ordering is used to construct the reduced-feature models with $n_x = 2$–11 in Fig. 2.

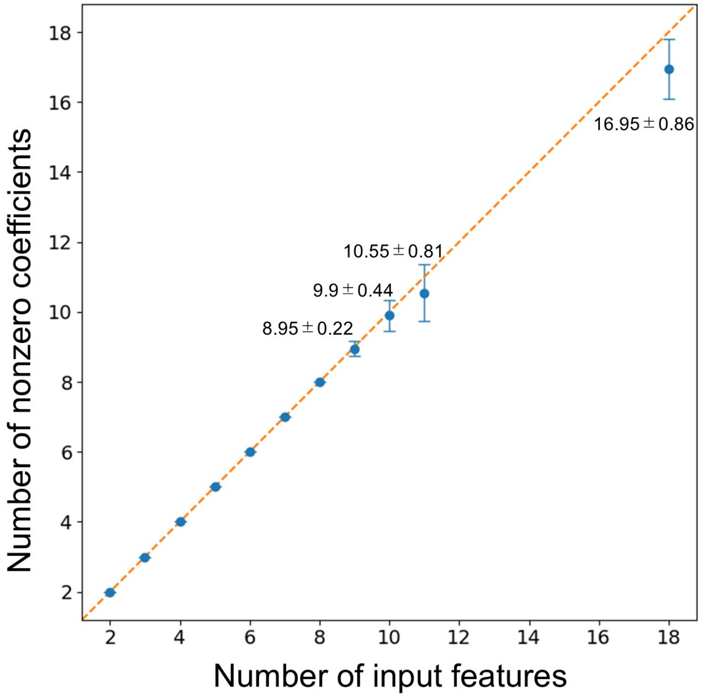

Fig. S8: Relationship between the number of input features and the number of nonzero (unshrunk) coefficients in LASSO regression models. Points and error bars represent the mean and one standard deviation obtained using 20 different data selections. For LASSO models with $n_x \leq 11$, the input feature subsets are determined based on the feature importance ranking derived from the absolute coefficients of the *11-feature* LASSO model. The diagonal line indicates the case where the numbers of input features and nonzero coefficients are equal.

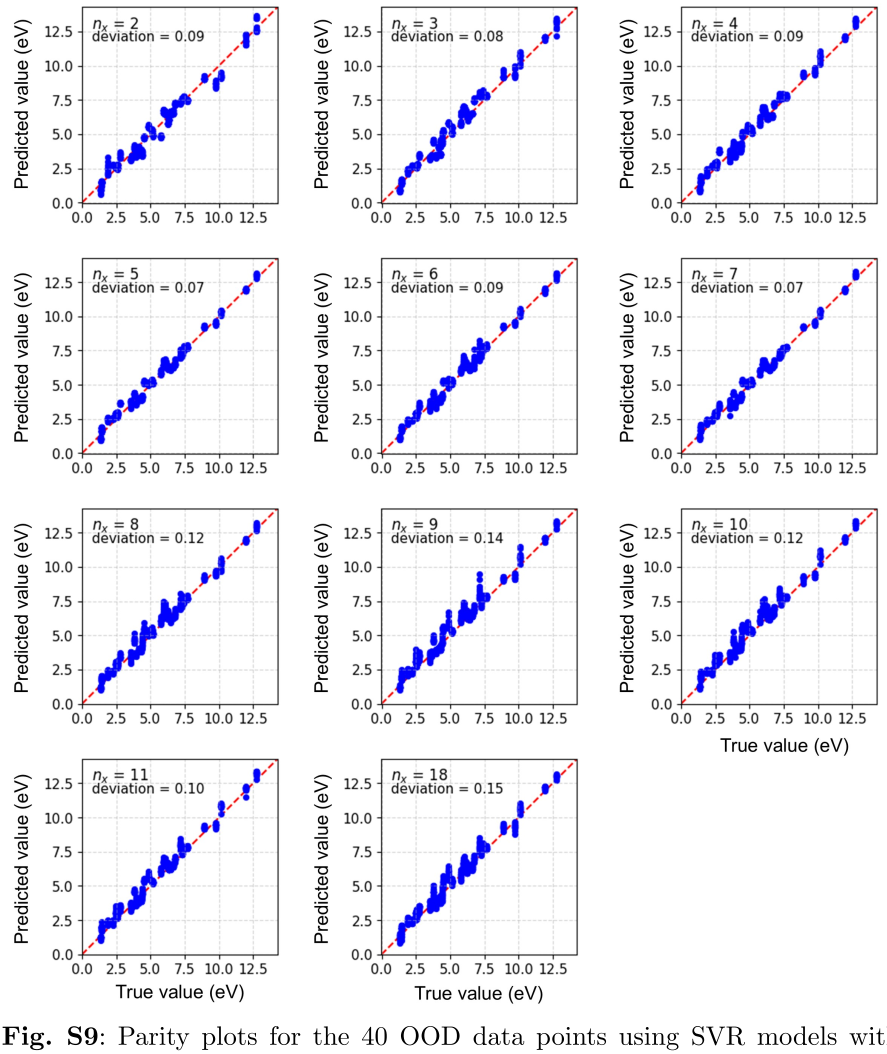

Fig. S9: Parity plots for the 40 OOD data points using SVR models with various numbers of features. Each dot represents the predicted values from the predictive models with 20 different data selections. The deviations of these predictions, obtained using equation (4), are also shown in each subplot.

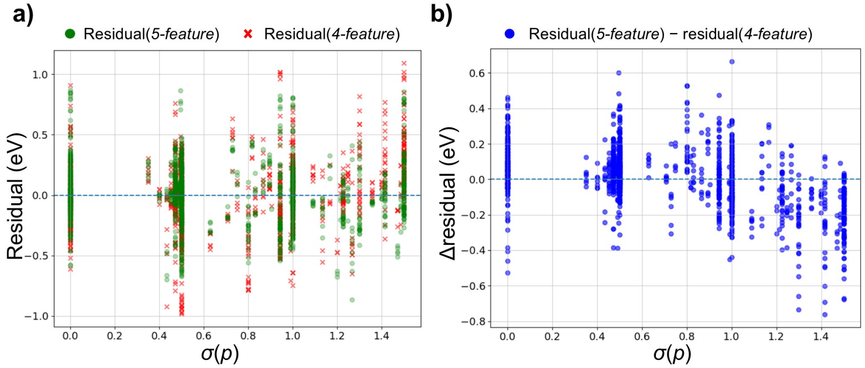

Fig. S10: (**a**) Distribution of prediction residuals for the in-domain test datasets (68 samples $\times$ 20 trials $=$ 1360 points) for the *4-feature* (red) and *5-feature* (green) SVR models as a function of $\sigma(p)$. The discrete values of $\sigma(p)$ reflect compositional regimes with different degrees of period mixing. At larger $\sigma(p)$, the *4-feature* model shows a consistent tendency toward positive residuals (overestimation), whereas inclusion of $\sigma(p)$ reduces this bias and shifts the residual distribution closer to zero. (**b**) Residual difference between the *5-feature* and *4-feature* models as a function of $\sigma(p)$. Negative values indicate reduction of the overestimation bias relative to the *4-feature* model. The bias reduction becomes more pronounced at larger $\sigma(p)$, indicating that $\sigma(p)$ modulates prediction behavior across compositional regimes rather than acting through direct linear correlation with the prediction objective.

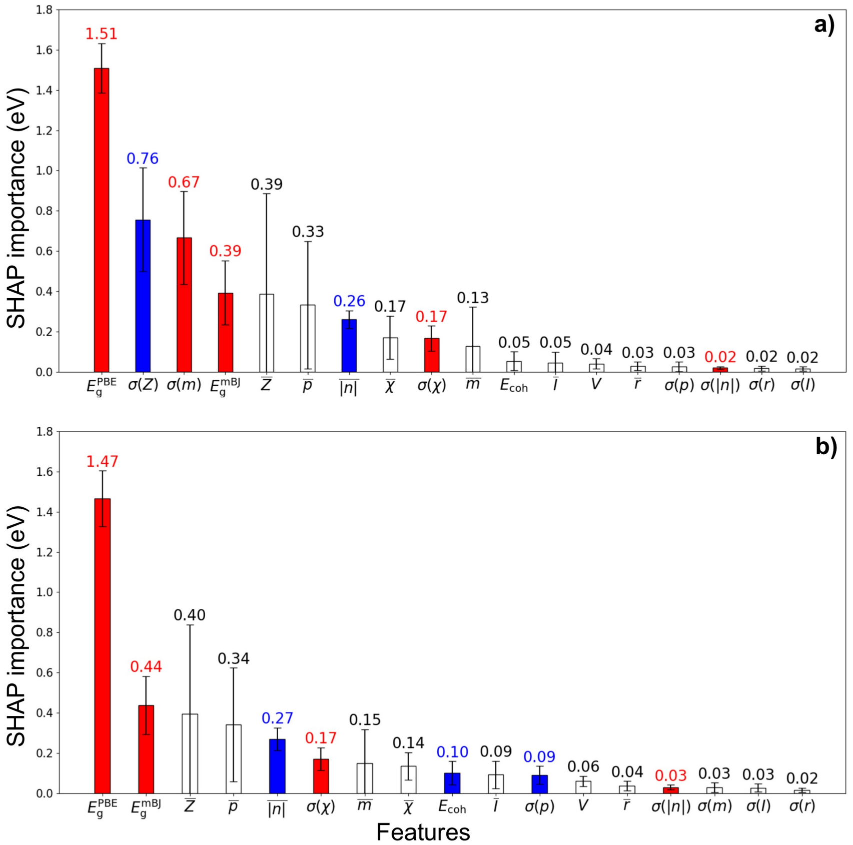

Fig. S11: SHAP importance scores for SVR models for predicting E_g^{GW} (**a**) with *18-feature set* and (**b**) with *17-feature set*, excluding $\sigma(Z)$. Detailed information relevant to most options such as error bars and colors for the bar graph are presented in Fig. 1.

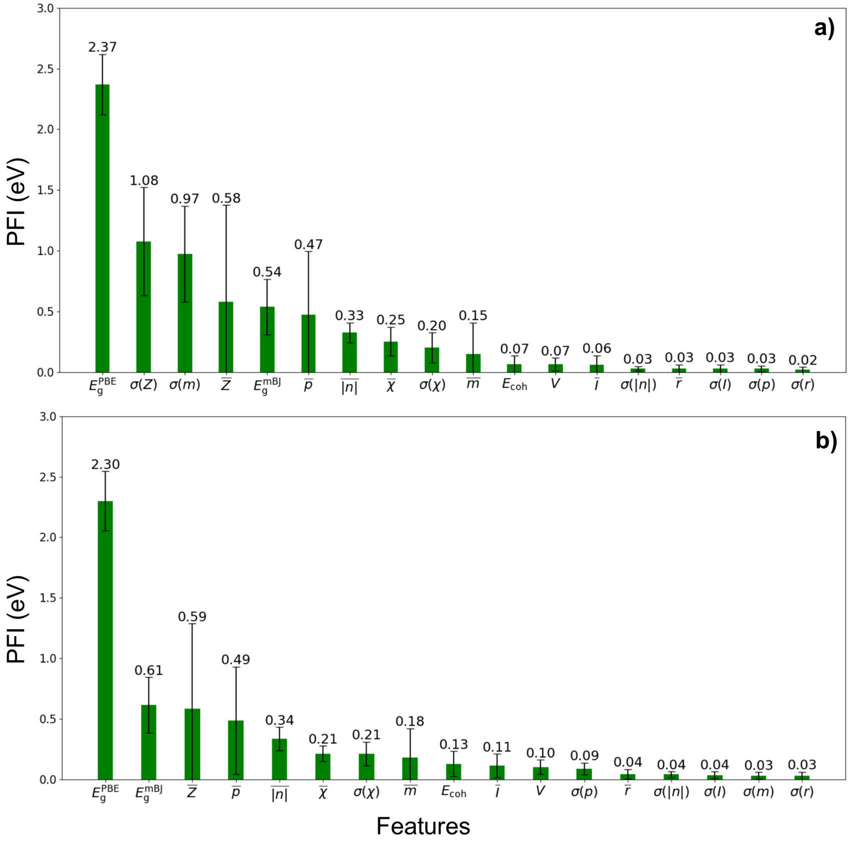

Fig. S12: PFI scores for SVR models for predicting E_g^{GW} (**a**) with *18-feature set* and (**b**) with *17-feature set*, excluding $\sigma(Z)$. Detailed information relevant to most options such as error bars for the bar graph are presented in Fig. 1.

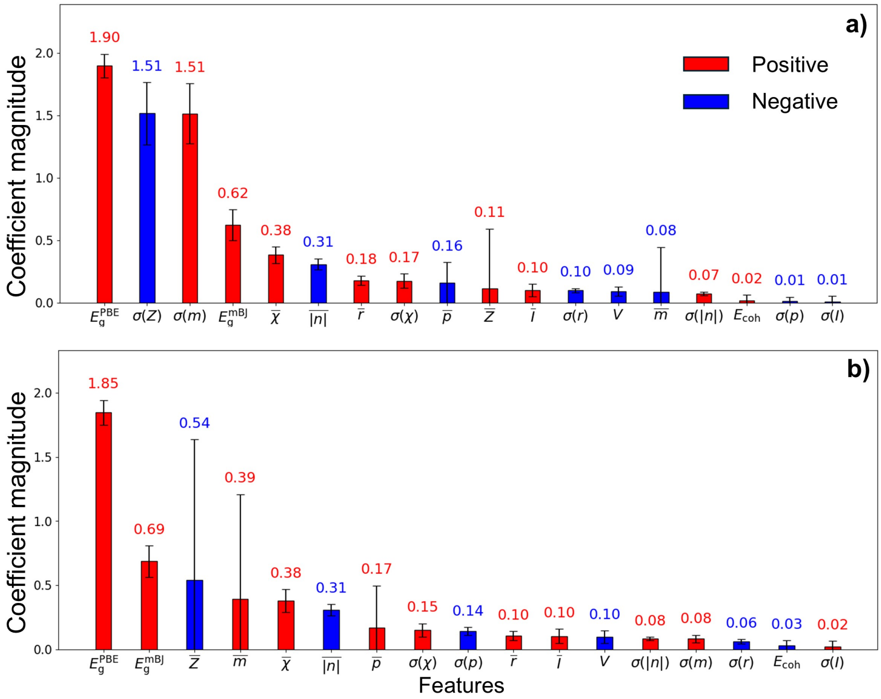

Fig. S13: Coefficient magnitudes for LASSO regression models for predicting $E_{\mathrm{g}}^{\mathrm{GW}}$ (**a**) with *18-feature set* and (**b**) with *17-feature set*, excluding $\sigma(Z)$. Detailed information relevant to most options such as error bars and colors for the bar graph are presented in Supplementary Fig. S7.

Table S1: Prediction objective (E_g^{GW}) and *18-feature set* for 40 inorganic compounds in the OOD dataset.

Material	MPD ID[a]	$E_g^{GW,(b)}$	$E_g^{PBE,(b)}$	$E_g^{mBJ,(b)}$	$V^{(c)}$	$E_{coh}^{(d)}$	$\lvert\bar{n}\rvert$	$\bar{Z}$	$\bar{p}$	$\bar{m}$
$Ba_2MgB_2O_6$	9259	7.2957	4.5121	8.1402	12.9682	−6.2480	2.1818	16.5455	2.8182	37.8710
K_2LiAlF_6	15549	11.9348	7.0616	11.1670	13.2148	−4.5830	1.2000	10.8000	2.5000	22.6175
$KAlSiO_4$	9480	7.6628	4.4997	7.2609	14.9860	−6.0202	2.2857	11.1429	2.5714	22.5949
KLi_6BiO_6	23582	3.9671	1.4444	2.4773	11.2193	−4.0971	1.7143	12.0000	2.4286	27.5813
$CaTiO_3$	4019	4.4942	2.4086	3.2974	11.0575	−6.6312	2.4000	13.2000	2.8000	27.1916
Ag_2GePbS_4	861942	2.5731	1.3148	1.9023	21.6331	−3.3590	2.0000	34.0000	4.0000	77.9763
$NaAlS_2O_8$	1210191	9.7422	5.7471	8.3629	11.5838	−5.0382	2.0000	10.0000	2.3333	20.1753
$AgAlS_2$	5782	3.6427	1.9515	3.0520	20.6793	−3.8678	2.0000	23.0000	3.5000	49.7453
$NbCu_3Se_4$	4043	2.4735	1.3589	1.5010	21.9054	−4.0599	2.0000	33.0000	4.1250	74.9235
Ba_2SrWO_6	18764	4.8743	2.8955	5.9543	15.7313	−6.1316	2.4000	27.2000	3.5000	64.2124
$RbSrCO_3F$	863745	7.4352	4.2828	7.7007	16.4389	−5.5448	2.0000	16.2857	2.8571	36.0139
$CaYAlO_4$	1227044	6.1561	3.6202	5.1058	11.0947	−6.5271	2.2857	14.8571	2.8571	31.4236
Sr_2ScGaO_5	1105158	5.1163	2.7354	4.7175	14.1686	−5.8407	2.2222	18.6667	3.1111	41.1021
Sr_2GaSbO_6	6304	4.3042	1.5673	3.0119	12.2842	−5.1016	2.4000	20.6000	3.1000	46.2713
$CdCu_2GeS_4$	13982	1.4490	0.4779	1.0420	19.4042	−3.4389	2.0000	25.2500	3.6250	55.0471
Sr_2NbInO_6	20222	6.0101	3.6961	4.5151	13.4476	−5.9338	2.4000	21.4000	3.2000	47.8970
$RbNaTiO_3$	556185	6.6509	3.5750	4.6381	14.6161	−5.3528	2.0000	15.6667	3.0000	34.0563
Li_2CdGeO_4	7688	5.1504	2.5202	4.6497	11.7934	−4.4159	2.0000	14.7500	2.6250	32.8801
$CeSiO_4$	10523	4.4220	1.6060	1.6553	12.6273	−6.9179	2.6667	17.3333	2.8333	38.7000
$CdAg_2I_4$	1025377	3.5544	1.7747	2.9001	36.1526	−1.9795	1.1429	50.5714	5.0000	119.3947
$Rb_2Li_2GeO_4$	8450	6.4371	3.5351	5.8293	15.0273	−4.2558	1.7778	16.0000	2.8889	35.7296
$SrTiO_3$	5229	3.7909	1.8501	2.7358	11.8274	−6.5718	2.4000	16.8000	3.0000	36.7000
$ZnCu_2SiTe_4$	1078498	1.3345	0.0622	0.6795	26.0467	−2.9551	2.0000	38.7500	4.3750	91.3709
SrAgTeF	1080438	2.7707	1.4149	2.5868	21.8604	−3.8344	1.5000	36.5000	4.2500	85.5215
Li_2ZnSnS_4	555186	4.1526	2.2812	3.2152	21.1218	−3.4531	2.0000	18.7500	3.1250	40.7980
VCi_3Te_4	991652	1.4492	0.5130	0.6041	24.4210	−3.4238	2.0000	39.7500	4.5000	93.9974
KNa_2CuO_2	545359	4.3044	1.6193	3.1185	16.5305	−3.3477	1.3333	14.3333	3.0000	30.1040
$KLiSO_4$	6179	8.9145	5.5435	9.1449	14.2480	−4.6714	1.7143	10.0000	2.4286	20.3106
Ba_2LaSbO_6	551269	6.7642	4.1321	5.9146	16.0099	−5.6548	2.4000	26.8000	3.5000	63.1304
$SrWO_4$	19163	7.1743	4.3956	5.3508	14.4879	−6.6017	2.6667	24.0000	3.1667	55.9117
$Y_2CN_2O_2$	546864	5.9604	4.0829	5.8054	13.6567	−7.0505	2.8571	16.2857	2.8571	35.6893
$LiCaAlF_6$	6134	12.7564	7.9256	10.9585	11.5957	−5.2724	1.3333	10.0000	2.3333	20.8952
$YScO_3$	768479	6.0666	3.8188	5.1118	13.3224	−7.3538	2.4000	16.8000	3.0000	36.3724
$NaZrCu_3Se_4$	1180089	2.6075	1.3386	2.1191	23.0723	−3.8742	1.7778	30.4444	4.0000	68.9658
Li_2MgCdP_2	1222661	2.2546	1.3722	2.2428	18.0372	−2.5509	2.0000	16.0000	3.0000	35.4473
$SrTaF_7$	555119	10.1503	5.5729	7.4006	13.0156	−5.6906	1.5556	19.3333	2.7778	44.6171
$CaMg_6ZnO_8$	1032894	6.3302	3.3602	5.2130	10.0093	−4.9620	2.0000	11.6250	2.6250	23.7061
VBO_4	754594	5.7642	2.7920	3.2228	14.7864	−6.5428	2.6667	10.0000	2.3333	20.9587
$Ca_2ZrTiSi_2O_{10}$	1227799	6.2472	3.2485	4.0439	12.0577	−6.7712	2.5000	13.1250	2.6875	27.2144
SrZnAsF	1080135	1.9016	0.5867	1.8223	18.7051	−3.4577	2.0000	27.5000	3.7500	61.7325

[a]From Materials Project Database. [Jain *et al.*, Appl. Mater. Phys. Lett. 1, 011002 (2013)]

[b]In the unit of eV.

[c]In the unit of Å^3/atom.

[d]In the unit of eV/atom.

Table S1: (Continued.)

Material	$\bar{r}$	$\bar{\chi}$	$\bar{I}$	$\sigma(\lvert\mathrm{n}\rvert)$	$\sigma(Z)$	$\sigma(p)$	$\sigma(m)$	$\sigma(r)$	$\sigma(\chi)$	$\sigma(I)$
$Ba_2MgB_2O_6$	1.8227	2.5282	10.5795	0.3857	18.6809	1.5266	47.0025	0.4314	1.0581	3.4646
K_2LiAlF_6	1.7980	2.8110	12.4596	0.6000	4.6861	0.8062	9.4045	0.4960	1.4465	6.0951
$KAlSiO_4$	1.8243	2.5843	10.4215	0.8806	4.0153	0.7284	8.4151	0.4315	1.0323	3.8299
KLi_6BiO_6	1.7757	2.0886	8.9775	1.0302	20.1282	1.1157	50.9776	0.3207	1.1945	4.0596
$CaTiO_3$	1.7960	2.5720	10.7591	0.8000	6.4000	0.9798	13.9271	0.3439	1.0767	3.5088
Ag_2GePbS_4	1.9438	2.2488	8.9886	0.8660	22.1980	1.1180	58.0362	0.1464	0.3355	1.3770
$NaAlS_2O_8$	1.6558	2.9350	11.7325	0.4082	3.0822	0.4714	6.3013	0.2237	0.8200	3.0098
$AgAlS_2$	1.8875	2.1750	8.5705	0.7071	13.9104	0.8660	33.6213	0.1295	0.4205	1.8758
$NbCu_3Se_4$	1.9575	2.1875	8.6185	1.2247	3.8079	0.3307	9.8554	0.0886	0.3740	1.1720
Ba_2SrWO_6	1.9150	2.5070	10.5691	1.2000	24.8548	1.8574	62.8484	0.5007	1.1631	3.7981
$RbSrCO_3F$	1.8929	2.6600	11.3442	0.9258	13.4453	1.3553	32.0184	0.5711	1.1886	4.4037
$CaYAlO_4$	1.7929	2.5129	10.3984	0.4518	10.6962	1.1249	24.9296	0.3475	1.0832	3.7183
Sr_2ScGaO_5	1.8444	2.4744	10.2268	0.4157	12.7976	1.2862	30.3908	0.4019	1.1052	3.7989
Sr_2GaSbO_6	1.8030	2.6400	10.7706	0.9165	16.0947	1.3748	38.9417	0.3867	1.0290	3.5723
$CdCu_2GeS_4$	1.9263	2.2275	9.2233	0.8660	10.8022	0.6960	27.0556	0.1433	0.3619	1.1966
Sr_2NbInO_6	1.8210	2.5920	10.5644	0.9165	16.6565	1.4697	39.7054	0.3974	1.0654	3.7508
$RbNaTiO_3$	1.9950	2.2683	9.4998	1.0000	10.7497	1.1547	25.6086	0.5533	1.1929	4.1906
Li_2CdGeO_4	1.7513	2.4275	10.2686	0.8660	15.2541	1.1110	36.0241	0.2588	1.0612	3.5299
$CeSiO_4$	1.7667	2.7967	11.3605	0.9428	18.3182	1.4625	45.5683	0.3609	0.9373	3.2807
$CdAg_2I_4$	2.0457	2.3129	9.4216	0.3499	2.8212	0.0000	8.7836	0.0789	0.4076	1.2669
$Rb_2Li_2GeO_4$	1.9878	2.1522	9.0566	0.9162	13.8724	1.2862	32.5143	0.5887	1.1996	4.2038
$SrTiO_3$	1.8320	2.5620	10.6755	0.8000	11.9063	1.2649	28.2960	0.4006	1.0914	3.6218
$ZnCu_2SiTe_4$	2.0338	1.9688	8.6296	0.8660	14.0601	0.6960	37.8793	0.0482	0.1519	0.6146
$SrAgTeF$	2.0325	2.2400	9.9259	0.5000	16.6508	1.2990	40.9260	0.3649	1.0963	4.4851
Li_2ZnSnS_4	1.8775	1.9863	8.6202	0.8660	14.2719	0.9270	34.0281	0.1293	0.6661	2.0971
VCi_3Te_4	2.0237	1.9663	8.2455	1.2247	12.3870	0.5000	33.8235	0.0495	0.1572	0.8210
KNa_2CuO_2	2.0483	1.9100	8.2636	0.4714	7.5203	0.8165	16.8242	0.4391	1.1395	3.9273
$KLiSO_4$	1.7786	2.5914	10.6521	0.4518	5.0990	0.7284	10.2983	0.4163	1.1094	3.8318
Ba_2LaSbO_6	1.8970	2.5570	10.6317	0.9165	23.0729	1.8574	57.8923	0.4888	1.1232	3.7671
$SrWO_4$	1.7917	2.7350	11.3386	1.4907	24.8998	1.6750	62.9092	0.3945	1.0203	3.2840
$Y_2CN_2O_2$	1.7829	2.5643	11.4285	0.6389	14.3797	1.3553	33.6809	0.3445	0.8945	3.4480
$LiCaAlF_6$	1.6433	3.0522	13.5586	0.6667	4.2687	0.6667	8.2960	0.2778	1.3229	5.4678
$YScO_3$	1.8060	2.5800	10.7266	0.4899	12.1885	1.2649	28.5607	0.3544	1.0542	3.5430
$NaZrCu_3Se_4$	1.9978	2.0178	8.2180	0.9162	7.6465	0.4714	18.5498	0.1377	0.5608	1.5681
Li_2MgCdP_2	1.8583	1.5567	8.0661	0.8165	15.1658	1.0000	35.8351	0.1470	0.5076	2.1237
$SrTaF_7$	1.6667	3.3678	15.0227	1.2571	21.0185	1.4741	52.7360	0.3734	1.1527	4.5114
$CaMg_6ZnO_8$	1.6788	2.3769	10.6456	0.0000	5.6665	0.6960	12.4570	0.2130	1.0693	3.0287
VBO4	1.6783	2.9050	11.5861	1.1055	5.9161	0.7454	13.5417	0.2281	0.7658	2.9084
$Ca_2ZrTiSi_2O_{10}$	1.7725	2.6919	11.1358	0.8660	8.5138	0.9823	19.5486	0.3310	0.9924	3.2472
$SrZnAsF$	1.9550	2.1900	10.5751	0.7071	11.0567	1.0897	25.9024	0.3659	1.1218	4.2638

Table S2: Sequential order of elimination of seven features from the pristine *18-feature set* prior to XML analyses.

# Features	r_p	r_s	Retained feature				Eliminated feature				p-value (pairwise	p-value (t-test	Max.
at test			Feature	RMSE (eV) [(a)]	r_p vs. E_g^{GW}	r_s vs. E_g^{GW}	Feature	RMSE (eV) [(a)]	r_p vs. E_g^{GW}	r_s vs. E_g^{GW}	t-test) [(bc)]	vs. pristine) [(d)]	VIF [(e)]
18	1.00	1.00	$\overline{Z}$	0.252 ± 0.024	−0.52	−0.55	$\overline{m}$	0.252 ± 0.025	−0.51	−0.53	0.761 [(c)]	0.091	448
17	1.00	0.99	$\sigma(m)$	0.245 ± 0.025	−0.37	−0.37	$\sigma(Z)$	0.246 ± 0.025	−0.35	−0.34	0.129 [(c)]	0.515	78.1
16	0.97	0.97	E_g^{PBE}	0.387 ± 0.042	0.98	0.98	E_g^{mBJ}	0.255 ± 0.033	0.98	0.98	1.9×10^{-9} [(b)]	0.096	74.7
15	0.97	0.96	$\overline{Z}$	0.262 ± 0.036	−0.52	−0.55	$\overline{p}$	0.263 ± 0.037	−0.52	−0.51	0.040 [(c)]	0.020	17.2
14	0.95	0.94	$\overline{\chi}$	0.268 ± 0.026	0.57	0.48	$\overline{I}$	0.251 ± 0.027	0.60	0.49	0.001 [(b)]	0.195	16.8
13	0.88	0.87	$\sigma(\chi)$	0.271 ± 0.028	0.80	0.81	$\sigma(I)$	0.258 ± 0.028	0.74	0.71	0.013 [(c)]	0.020	8.1
12	0.77	0.80	$\overline{Z}$	0.252 ± 0.027	−0.52	−0.55	V	0.255 ± 0.025	−0.22	−0.25	0.514 [(c)]	0.063	7.9
11	0.73	0.74	E_g^{PBE}	0.960 ± 0.131	0.98	0.98	$\sigma(\chi)$	0.289 ± 0.022 [(d)]	0.80	0.81	2.4×10^{-15}	1.1×10^{-7}	4.9

[a] The RMSE obtained using the predictive model when this feature is removed. ± indicates one standard deviation across the predictive models with 20 different data selections.

[b] The elimination is based on a statistically significant increase (99% confidence level) in the prediction error (p-value < 0.01).

[c] The elimination is based on lower correlation with the prediction objective (E_g^{GW}) as no significant error difference is observed (p-value > 0.01).

[d] At each elimination step, the prediction error of the reduced feature set is compared with that of the pristine model using a paired t-test. If the removal of a feature does not result in a statistically significant increase in prediction error (p-value > 0.01), the elimination process proceeds to the next step. If a statistically significant increase is observed (p-value < 0.01), the elimination process is terminated. Consequently, the feature set is reduced to 11 features.

[e] The maximum variance inflation factor (VIF) at each elimination step is additionally reported to illustrate the progressive reduction of multicollinearity. The reported value corresponds to the maximum VIF after the removal of one feature at each stage. For the original 18-feature set, the maximum VIF is 8135.

Table S3: Prediction errors for test dataset using SVR models with different numbers of features. ± indicates one standard deviation across the predictive models with 20 different data selections.

Number of	In-domain			OOD		
features	RMSE (eV)	MAE (eV)	R^2	RMSE (eV)	MAE (eV)	R^2
18	0.247 ± 0.019	0.184 ± 0.013	0.993 ± 0.001	0.460 ± 0.053	0.376 ± 0.042	0.980 ± 0.004
11	0.255 ± 0.025	0.188 ± 0.018	0.993 ± 0.001	0.457 ± 0.037	0.373 ± 0.026	0.983 ± 0.003
10	0.254 ± 0.023	0.187 ± 0.015	0.993 ± 0.001	0.473 ± 0.069	0.382 ± 0.049	0.981 ± 0.007
9	0.253 ± 0.021	0.186 ± 0.014	0.993 ± 0.001	0.483 ± 0.103	0.386 ± 0.065	0.979 ± 0.010
8	0.266 ± 0.031	0.195 ± 0.017	0.992 ± 0.002	0.398 ± 0.053	0.316 ± 0.037	0.982 ± 0.004
7	0.260 ± 0.025	0.189 ± 0.016	0.993 ± 0.002	0.335 ± 0.019	0.279 ± 0.014	0.987 ± 0.001
6	0.259 ± 0.026	0.189 ± 0.016	0.993 ± 0.002	0.354 ± 0.039	0.292 ± 0.032	0.985 ± 0.003
5	0.254 ± 0.020	0.191 ± 0.014	0.993 ± 0.001	0.348 ± 0.014	0.287 ± 0.012	0.986 ± 0.000
4	0.289 ± 0.021	0.217 ± 0.015	0.991 ± 0.002	0.427 ± 0.027	0.355 ± 0.021	0.982 ± 0.001
3	0.312 ± 0.032	0.234 ± 0.023	0.989 ± 0.003	0.486 ± 0.021	0.393 ± 0.018	0.977 ± 0.002
2	0.453 ± 0.045	0.313 ± 0.033	0.978 ± 0.004	0.483 ± 0.019	0.388 ± 0.020	0.969 ± 0.002
